# Cavernous malformation hemorrhage due to trans-mural pressure alterations after cerebrospinal fluid diversion: a case report

**DOI:** 10.1186/s12883-020-01714-3

**Published:** 2020-04-13

**Authors:** Benjamin R. Hartley, Corinne Birnbaum, Caitlin E. Hoffman

**Affiliations:** 1 Department of Neurosurgery, Weill-Cornell College of Medicine/New York Presbyterian Hospital, 525 East 68th Street, Box, New York, NY 99 USA; 2 Columbia University, New York, NY USA

**Keywords:** Cavernous malformation, Hemorrhage, Trans-mural pressure, Cerebrospinal fluid

## Abstract

**Background:**

Cavernous malformations are rare cerebral pseudo-vascular lesions with annualized bleeding rates of 0.5–3% in most studies. Of the various explored risk factors for bleeding to date, only prior hemorrhage has shown significant correlation.

**Case presentation:**

In this case, we describe a 65-year old man with a peri-ventricular atrial cavernous malformation that hemorrhaged after CSF diversion via ventriculoperitoneal shunting. Serial imaging showed that bleeding continued until the shunt was revised with a programmable valve set at maximum resistance with the addition of a gravitational unit, thereby lowering the trans-mural pressure differential across the cavernous malformation.

**Conclusions:**

Given that other vascular lesions are subject to hemorrhage from alterations in trans-mural pressure dynamics, we hypothesize that cavernous malformations are similarly affected by trans-mural pressure gradients as they are composed of primitive vascular elements. This hypothesis is corroborated by the temporal correlation of interventions, imaging, and exam findings in the present case, and suggests a potentially important risk factor for hemorrhage in CM patients that affects prognostication and management.

## Background

Cavernous malformations (CM) are rare cerebral abnormalities composed of low-flow, endothelium-lined, thin-walled caverns filled with blood at various stages of thrombosis and organization, separated by a collagenous stroma but devoid of mature vessel wall elements. Usually they become symptomatic due to interval hemorrhage and associated sequelae [1–3]. Most studies determined annualized bleeding rate as 0.5–3% [4–7]. The effects of intracranial pressure (ICP), transmural pressure, or alteration in CSF flow dynamics on CM hemorrhage are poorly understood. Explored potential risk factors for hemorrhage include sex, age, lesion location, size, trauma, perfusion, and prior hemorrhage [3, 8–17]. Of these, prior hemorrhage and brainstem location are the only proven risk factors. To date, neither causality nor correlation have been established between ICP, transmural pressure changes, or CSF flow dynamics and CM hemorrhage. Herein, we discuss the case of a 65-year old man with a history of multiple hemorrhages related to a left peri-atrial CM and resultant hydrocephalus who experienced lesion-associated intraventricular hemorrhage (IVH) following placement of a ventriculoperitoneal shunt (VPS). The significance of this finding informs additional potential risk factors for hemorrhage, patient counseling, and interventional planning.

## Case presentation

**Fig. 1:**
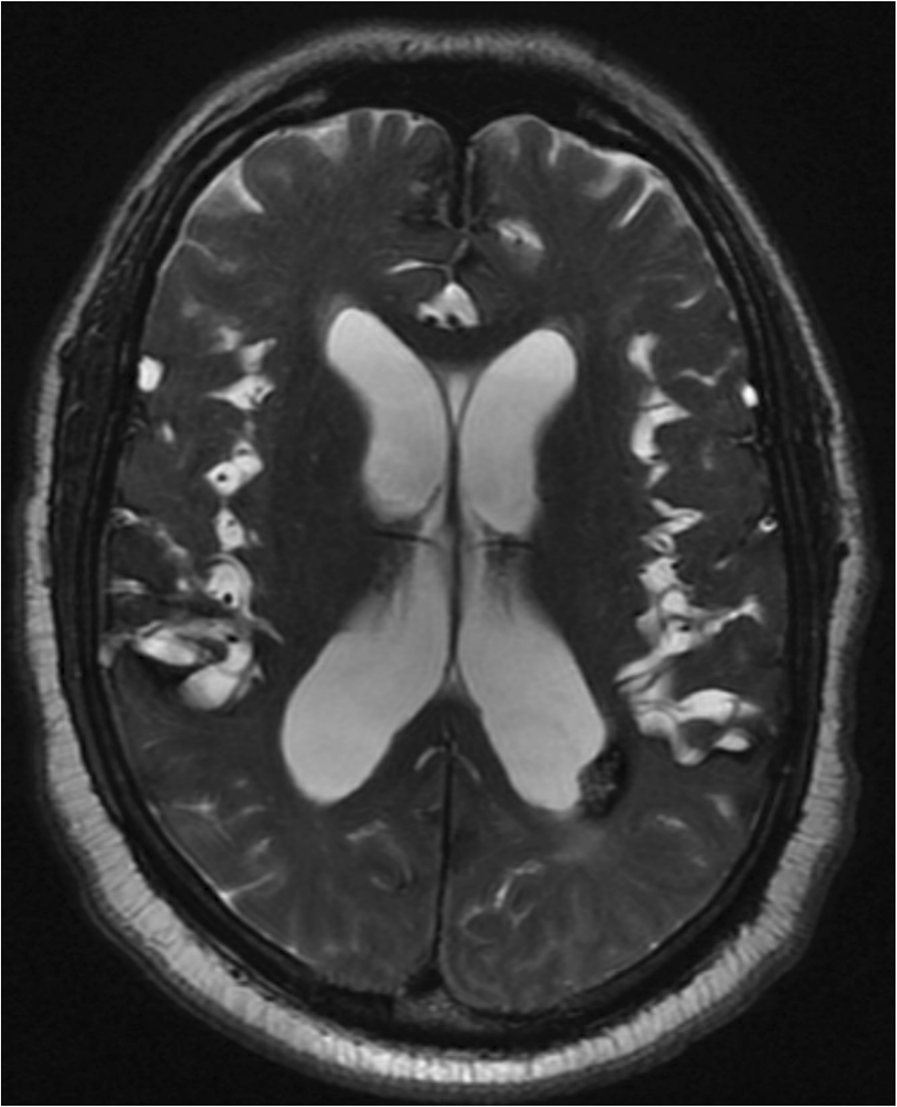
T2 weighted MRI brain without contrast on admission, showing peri-atrial cavernous malformation and hydrocephalus

**Fig. 2:**
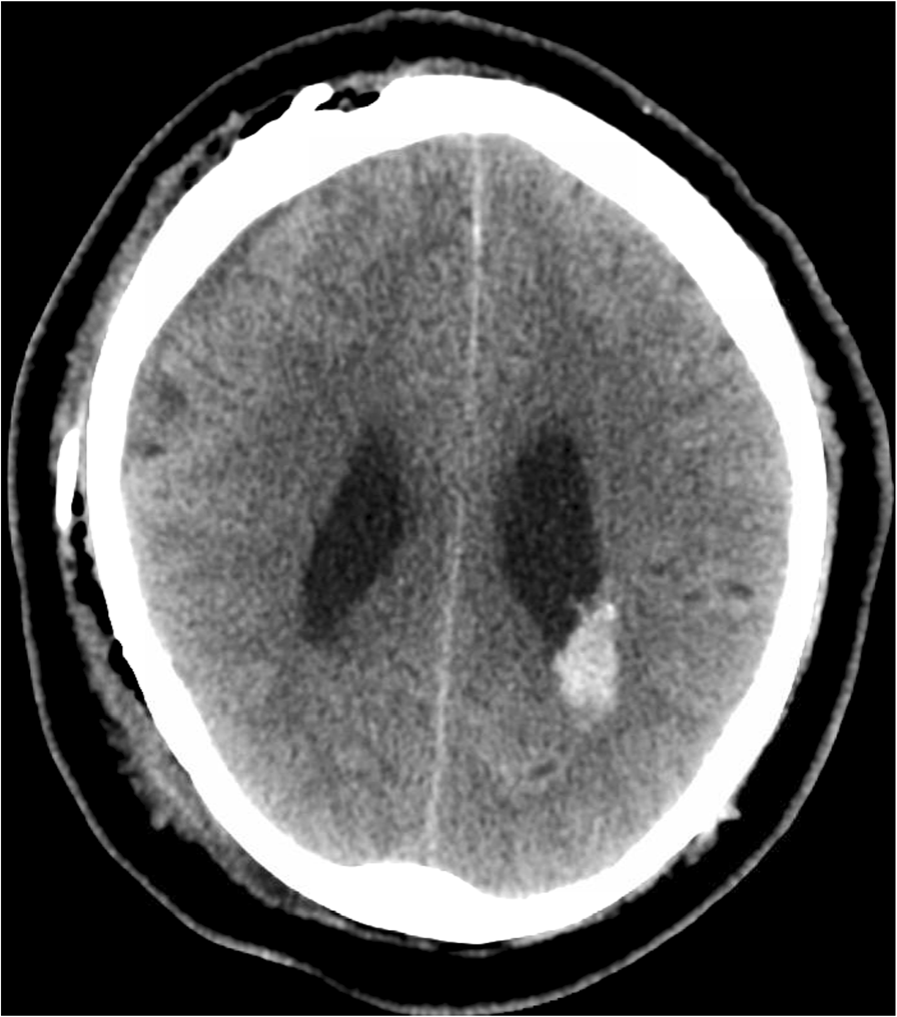
CT head post op day 1 after ventriculoperitoneal shunt, showing interval hemorrhage of cavernous malformation

**Fig. 3:**
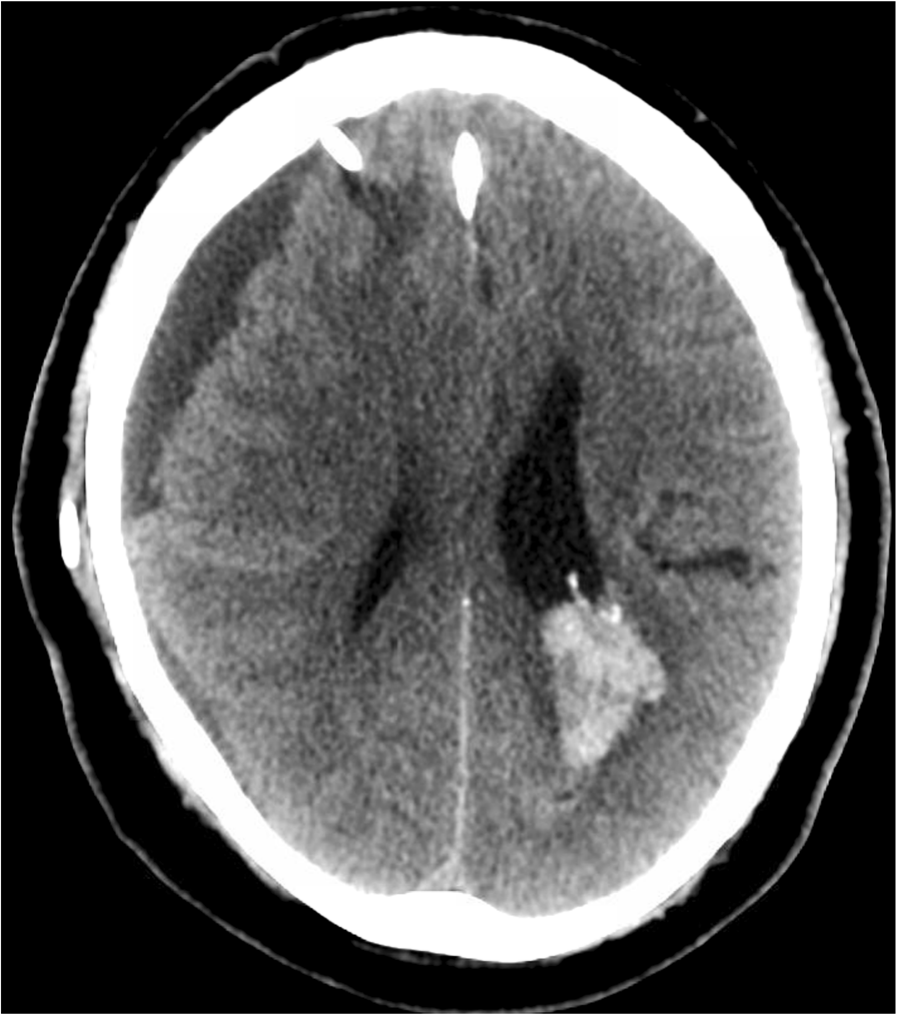
CT head post op day 9, showing interval expansion of hemorrhage and subdural hygroma

**Fig. 4:**
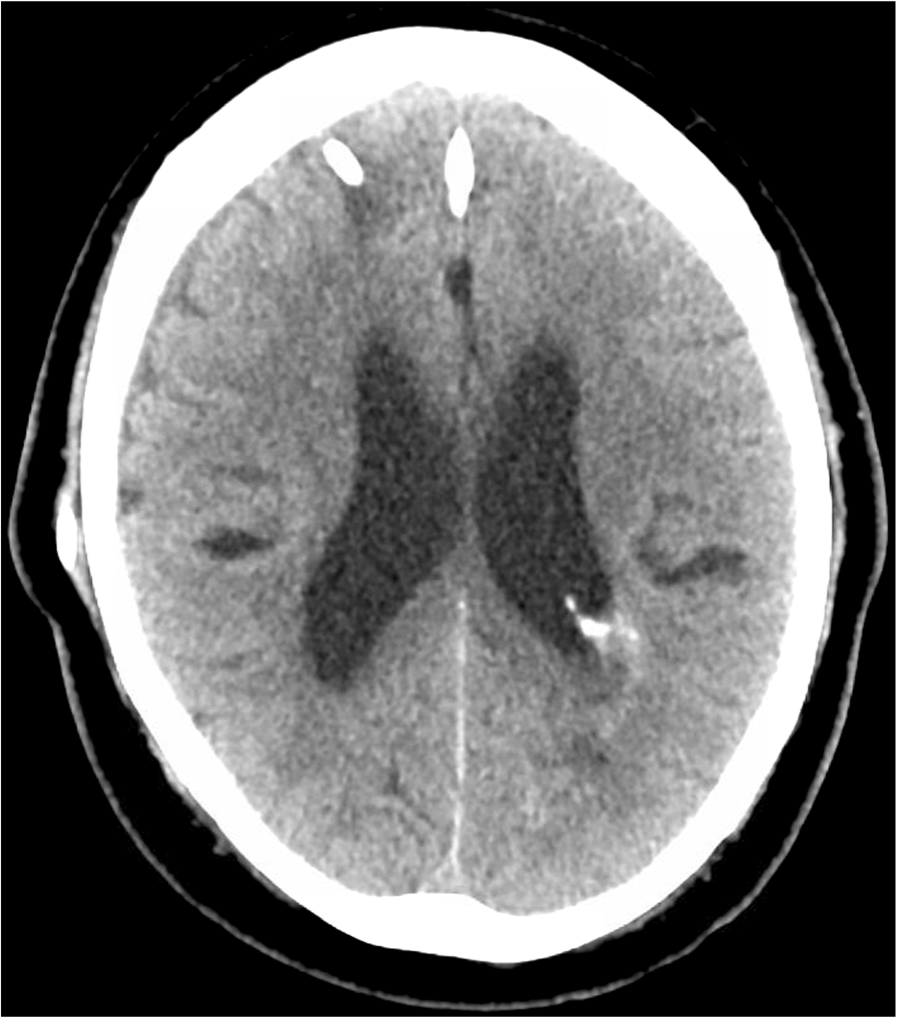
CT head post op day 25 after revision, showing resolution of hygroma and CM-related hemorrhage

A 65-year old man with known left atrial CM measuring 1.3 cm in largest dimension, and a history of related hemorrhages of this lesion resulting in IVH and subarachnoid siderosis presented with headaches, papilledema, and hydrocephalus (Fig. 1). Notably, previous hemorrhages of this lesion, of which there were four reported over 15 years, were relatively mild, and did not result in major morbidity or require treatment. A VPS with a medium pressure valve was placed, and post-operative head CT demonstrated interval hemorrhage from the CM with associated IVH (Fig. 2), which progressively expanded on interval imaging (Fig. 3). The VPS catheter tip terminated in the right foramen of Monroe (not shown). Importantly, laproscopy was not utilized for abdominal access, nor was the abdomen insufflated, and only 3 ml of CSF were removed intraoperatively. Of note, the patient developed a subdural hygroma on post op day 10, further suggesting a significant focus of relatively negative intracranial pressure as a result of VPS placement. Based on the assumption that the IVH was secondary to alterations in transmural pressure across the CM, a programmable valve set at maximal resistance (Medtronic Strata, 2.5), as well as an anti-siphon device (Codman, SiphonGuard) were placed to decrease this pressure gradient, which should also prevent future hemorrhage via the same mechanism. Interval imaging showed resolution of active bleeding (Fig. 4). Ultimately the patient was discharged uneventfully with complete remission of presenting symptoms. Of note, the patient has one additional, smaller, left temporal CM with an associated developmental venous anomaly (DVA), but there is no history of hemorrhage of this lesion. The patient declined genetic testing for familial CM syndrome.

## Discussion and conclusions

Causative factors of CM-associated hemorrhage are currently under investigation and remain poorly understood, though many potential mechanisms have been suggested. The current data, including original studies, meta-analyses, and literature review suggest that only prior hemorrhage and brainstem location prognosticated significant bleeding risk [7, 11, 17–21]. To date, no studies have directly investigated the relationship between ICP, transmural pressure, or CSF flow dynamics and CM hemorrhage. Some have suggested a correlation with hydrocephalus in general, though establishing causality in these cases is difficult as intracranial hemorrhage in general can pre-dispose to hydrocephalus [4, 22].

In the present case, the patient presented with hemorrhage-related hydrocephalus from a known periventricular cavernous malformation; VPS placement on the contralateral side resulted in CM hemorrhage, which stabilized once CSF diversion, and therefore transmural pressure gradient, were decreased. Importantly, the abdomen was not insufflated during surgery, which could potentially increase venous pressure and lead to hemorrhage, nor was significant CSF removed, which could cause a transient, extreme transmural pressure gradient across the lesion. This case suggests that placement of the VPS and lowering the ICP increased transmural pressure differential, which disturbed the internal flow dynamics of the CM leading to hemorrhage; increasing the valve setting, and by proxy the ICP around the CM, stabilized the hemorrhage. The apparent fragility of these pseudo-vascular lesions, and thereby their susceptibility to relatively small changes in transmural pressure differential, may be suggested by the finding that they are composed of immature, disorganized proto-endothelium [20]. This hypothesis is further corroborated by cerebral and aortic aneurysm and arteriovenous malformation studies, which suggest that alterations in transmural pressure, such as by CSF drainage, blood pressure changes, and even atmospheric pressure, increases the risk of hemorrhage from these lesions [23–26]. These studies utilized both model and in vivo human and animal data to demonstrate that pressure differentials across luminal walls of aneurysmal lesions influence hemorrhage risk. While one may not anticipate that a CM is subject to these same factors given that they are low-flow “vascular” lesions without truly differentiated vascular elements [3, 20], the temporal correlation of interventions, imaging findings, and symptoms in this case suggests possible causality. It is also of note that the peri-ventricular location of this CM may have pre-disposed to greater risk of hemorrhage from alterations in CSF flow dynamics and ICP.

While causality is difficult to establish, the present case suggests correlation between alteration in CSF flow dynamics or ICP and cavernous malformation hemorrhage, strengthened by the fact that current data supports a causal link between CSF flow dynamics, transmural pressure, and hemorrhage in other intracranial vascular lesions, and that changes in these parameters resulted in hemorrhage and subsequent stabilization. Future directions include prospective studies to establish causality between ICP changes via CSF drainage and CM hemorrhaging, and studies of vascular flow dynamics of cavernous malformations in relation to transmural pressure.

## Data Availability

The datasets generated and/or analyzed during the current study are not publicly available as they are protected by patient privacy laws and regulations, but de-identified data are available from the corresponding author on reasonable request.

## Abbreviations

CM: Cavernous malformations
ICP: intracranial pressure
IVH: intraventricular hemorrhage
VPS: ventriculoperitoneal shunt
